# Does Zika virus infection affect mosquito response to repellents?

**DOI:** 10.1038/srep42826

**Published:** 2017-02-16

**Authors:** Walter S. Leal, Rosângela M. R. Barbosa, Fangfang Zeng, Gabriel B. Faierstein, Kaiming Tan, Marcelo H. S. Paiva, Duschinka R. D. Guedes, Mônica M. Crespo, Constância F. J. Ayres

**Affiliations:** 1 Department of Molecular and Cellular Biology, University of California-Davis, Davis, CA 95616, USA; 2 Department of Entomology, Centro de Pesquisas Aggeu Magalhães, Fundação Oswaldo Cruz, Campus da Universidade Federal de Pernambuco, Recife, PE, 50.740-465, Brasil; 3 Universidade Federal de Pernambuco, Centro Acadêmico do Agreste - Rodovia BR-104, Km 59 - Nova Caruaru, Caruaru - PE – CEP: 55002-970, Brasil

## Abstract

The World Health Organization (WHO) recommends that people travelling to or living in areas with Zika virus (ZIKV) outbreaks or epidemics adopt prophylactic measures to reduce or eliminate mosquito bites, including the use of insect repellents. It is, however, unknown whether repellents are effective against ZIKV-infected mosquitoes, in part because of the ethical concerns related to exposing a human subject’s arm to infected mosquitoes in the standard arm-in-cage assay. We used a previously developed, human subject-free behavioural assay, which mimics a human subject to evaluate the top two recommended insect repellents. Our measurements showed that DEET provided significantly higher protection than picaridin provided against noninfected, host-seeking females of the southern house mosquito, *Culex quinquefasciatus*, and the yellow fever mosquito, *Aedes aegypti.* When tested at lower doses, we observed a significant reduction in DEET-elicited protection against ZIKV-infected yellow fever mosquitoes from old and recent laboratory colonies. The reduction in protection is more likely associated with aging than the virus infection and could be compensated by applying a 5x higher dose of DEET. A substantial protection against ZIKV-infected and old noninfected mosquitoes was achieved with 5% DEET, which corresponds approximately to a 30% dose in the conventional arm-in-cage assays.

The Zika virus (ZIKV) was isolated from a sentinel rhesus monkey almost seven decades ago during a long-term research program sponsored by the Rockefeller Foundation and aimed at unravelling the cycle of sylvan yellow fever virus in Uganda^1^. Among the seven new viruses discovered in the Yellow Fever Research Institute at Entebbe, the West Nile Virus (WNV), became notorious earlier on, particularly for causing “seasonal epidemics” in North America and being the most common cause of neuroinvasive (encephalitis, meningitis, or acute flaccid paralysis) arboviral diseases in the United States of America^2^. Meanwhile, no one paid much attention to ZIKV, because up to an outbreak that occurred in the Yap Island in 2007 only 14 human cases had been identified^3^. On Yap Island, Federal State of Micronesia, it was estimated that 73% of residents 3 years of age or older have been infected with ZIKV^4^. Here, it was observed that in addition to rash, conjunctivitis, and arthralgia, Zika infection might lead to Guillain-Barré syndrome^5^. It was in Brazil in 2015, however, that ZIKV gained notoriety particularly given its teratogenic effect^6^, which led the World Health Organization (WHO) to declare on February 1, 2016 ZIKV an international public health emergency; as of November 18, 2016, WHO updated the ZIKV status to a “significant enduring public health challenge requiring intense action.” From Brazil, ZIKV disseminated so rapidly that one year after its isolation from Brazilian patients^7^,^8^, ZIKV reached as many as 40 countries in the Americas, including the United States. At the time of this writing, 4,575 ZIKV infections have been reported in the United States, mostly travel-associated cases, in addition to 185 locally transmitted by mosquito bites, with Florida leading the statistics on autochthonous transmission and New York reporting as many as 942 travel-associated ZIKV cases. In US territories, more than 33,000 cases have been reported, most of them being locally transmitted^9^. Therefore, people travelling to or living in ZIKV epidemic or outbreak areas are strongly advised to take prophylactic measures to avoid acquiring and further disseminating the virus. Without a ZIKV vaccine, the main recommendations by WHO are wearing clothes that cover as much of the body as possible, using physical barriers such as window screens, sleeping under mosquito nets, and using insect repellents for protection against mosquito bites.

Chemicals used to reduce mosquito bites are not only repellents sensu stricto, i.e., those compounds that cause the responder to steer away from the source, but also excitorepellents or irritants, i.e., chemicals eliciting increasing locomotion activity after an insect makes contact with the source^10^. From a strict mechanistic perspective, these two groups should be named noncontact and contact disengagents, respectively^11^. From a pragmatic viewpoint, it would suffice to consider that spatial repellents are odorants and contact repellents are tastants, i.e., their modes of action are “smell and avoid” and “taste and walk away,” respectively. Although contact repellents (contact disengagents) are invaluable in preventing mosquito bites, spatial repellents are preferred for skin application given that viruses may be transmitted while a mosquito is probing the skin prior to initiating a blood meal^12^. Additionally, virus replication in the salivary glands may affect the gustatory system^13^ and, consequently, reduce contact repellency sensitivity^14^.

We then raised the question whether ZIKV infection would alter mosquito responses to insect repellents recommended for ZIKV prevention^15^. Efficacy of mosquito repellents for human skin is measured according to guidelines by the Environmental Protection Agency (EPA)^16^ and WHO^17^. In short, test repellents are applied directly to the arm of a human volunteer, which is then inserted into a cage housing healthy, host-seeking female mosquitoes to determine landing and/or probing activity. Although alternative methods that prevent direct contact between the mosquito and the subject’s arm have been designed^18^,^19^, legitimate ethical concerns preclude measuring repellency with EPA and WHO standard arm-in-cage protocols when using infected mosquitoes. We applied a previously developed two-choice human subject-free, noncontact repellency assay^20^ to test protection of commercial repellents against ZIKV-infected mosquitoes. Here, we report that the oldest synthetic repellent on the market, DEET (*N,N*-diethyl-3-methylbenzamide), has a higher protection rate as a spatial repellent against both the southern house mosquito*, Culex quinquefasciatus*, and the yellow fever mosquito, *Aedes aegypti*, than its more modern counterpart, picaridin (Icaridin, Bayrepel^®^, butan-2-yl 2-(2-hydroxyethyl)piperidine-1-carboxylate). Additionally, we show both laboratory and field populations of the yellow fever mosquito from Brazil appear to have somewhat reduced responses to repellents at the age that they are competent for ZIKV transmission than younger, healthy mosquitoes used for standard efficacy testing. Our findings suggest, for protection against ZIKV, higher doses of DEET-based repellents should be applied.

## Results and Discussion

**Figure 1:**
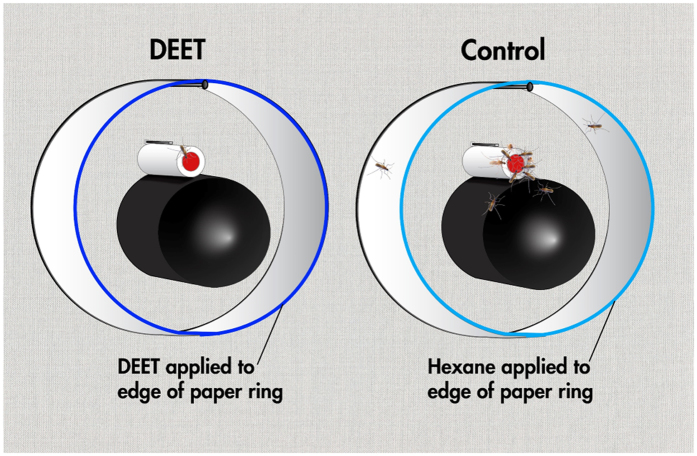
Diagrammatic representation of the human subject-free, surface landing, and feeding assay. The ends of two internally painted Dudley bubbling tubes extend from a wooden board. The tubes were connected behind the board to a water bath and surrounded by filter paper rings in the behavioural arena. Blunted tips of stainless steel needles placed on the top of each tube served not only to deliver CO_2_, but also to retain dental cotton rolls. The rolls were loaded with defibrinated sheep blood on the circular surface facing the mosquito cage (not seen). Hexane (200 μl) was applied to the edge of one ring (control), and the same volume of a solution of repellent (e.g., DEET) to another ring (test). After allowing evaporation for at least 3 min, the rings were placed in the arena and secured by insect pins. Graphics created by Steven Oerding, IET Academic Technology Services, University of California-Davis.

Our surface landing and feeding assay (Fig. 1) differs in multiple features from the arm-in-cage standard protocol^16^,^17^. First and foremost, it allows measurement of repellency behaviour in the absence of a human subject, which is invaluable when measuring behaviour of infected mosquitoes. Secondly, it evaluates only spatial repellency given that the blood and repellent sources are spatially separated; thus, to reach a blood source, no direct contact between the repellent and the gustatory system is needed. The chemical curtain generated by the vapour phase of a test repellent applied to a filter paper ring surrounding the attractant source causes the responder to steer away from airborne chemicals (spatial repellency). Lastly, this two-choice assay allows simultaneous assessment of repellency and mosquito avidity for a blood meal based on the responses to the control side of the arena. This assay thus reduces/eliminates the possibility of false-positives when testing one at a time in no-choice assays like the arm-in-cage. It is, however, a more rigorous measurement of repellency given that the mosquito’s olfactory system must be activated at least 3–5 cm from the source to elicit repellency.

**Figure 2:**
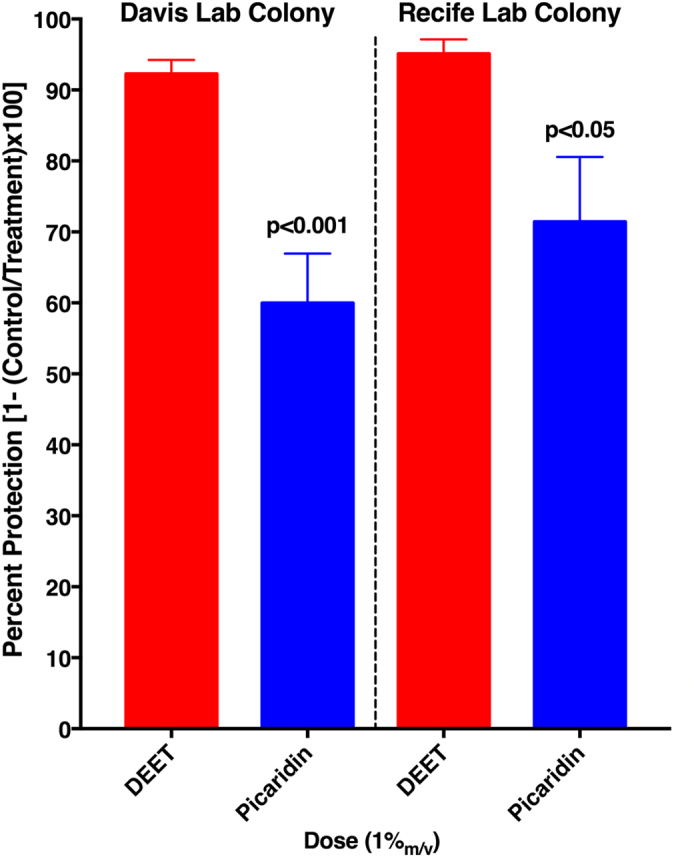
Repellency activity elicited by DEET (red) or picaridin (blue) from two geographically different laboratory colonies of the southern house mosquito. Picaridin at 1% provided significantly lower protection than DEET provided at the same dose (two-tailed t-test, Davis Lab colony, p = 0.0002, n = 12; Recife Lab colony, p = 0.0301, n = 6). The data are expressed as mean ± SEM.

Using these surface-landing and feeding assays, we first compared repellency elicited by DEET or picaridin on host-seeking females of the southern house mosquito (common mosquito), *Culex quinquefasciatus.* Our initial test dose (1%) is close to what is found in many commercial products in the US and overseas, e.g., OFF! Familycare^®^ unscented, Cutter Skinsations^®^, Cutter All Family^®^, all 7% DEET (US); Super Repelex^®^, 6.7% DEET; Xo Inseto!^®^, 7.5% DEET (Brazil), just to cite a few. A dose of 1% is equivalent to a dose of approximately 6.3% (63 mg of a test repellent) applied directly to the skin in an arm-in-cage test. In the surface-landing and feeding assays, an aliquot of 200 μl of a 1%_m/v_ solution (2 mg of a test repellent) was applied to a filter paper area of approximately 19 cm^2^ (perimeter, 24 cm; width, 7.91 ± 1.19 mm; mean ± SEM, *n* = 28 measurements), i.e., approximately 0.105 mg/cm^2^, whereas in the arm-in-cage experiments 1 ml of a 6.3% dose, would be applied to approximately 600 cm^2^ of the forearm skin, i.e., approximately 0.105 mg/cm^2^. In behavioural tests with 5- to 7-day-old host-seeking females from the Davis colony, DEET at a 1% dose provided significantly higher protection (*n* = 12; *P* = 0.0002, unpaired, two-tailed t-test) than picaridin provided at the same dose (Fig. 2). Similar results were observed with a laboratory colony initiated from mosquitoes collected in Recife, Brazil, and maintained at FIOCRUZ-PE since 2009. Protection elicited by picaridin at a 1% dose against 5- to 7-day-old host-seeking females of the Recife colony were not significantly different from the protection observed with the Davis colony (Mann Whitney two-tailed test, *P* = 0.301). DEET at 1% elicited higher protection than picaridin (*n* = 6; *P* = 0.0043, Mann-Whitney two-tailed test) (Fig. 2), similarly with what has been observed with the Davis laboratory colony.

Because picaridin was developed at the end of the last century, there have been many studies comparing DEET and picaridin formulations. A review of these efficacy tests^21^ concluded based on peer-reviewed literature that DEET at a concentration of 20% or greater had the greatest efficacy against *Aedes* mosquitoes, and that *Culex* mosquitoes are easier to repel, with all four repellents reviewed (DEET, picaridin, IR3535, and Citridora [p-menthane-3,8-diol]) providing good protection^21^. To the best of our knowledge, there were only two studies comparing DEET with picaridin in solutions rather than commercial formulations. One of these studies concluded that picaridin was significantly less effective than DEET in reducing *Ae. aegypti* bites^22^, whereas the other showed the two repellents were not significantly different for protection against *Anopheles gambiae*, but picaridin was 1.5x more potent than DEET for *Ae. aegypti*^23^.

**Figure 3:**
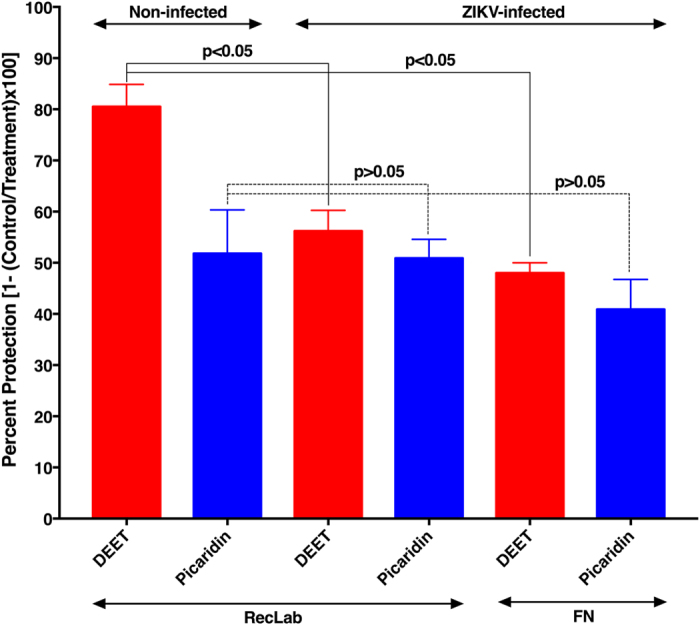
Protection elicited by DEET or picaridin against noninfected and ZIKV-infected yellow fever mosquitoes. Two groups of mosquitoes were tested. RecLab - a 10-year-old laboratory colony established with mosquitos collected in Recife, Brazil, and FN - a laboratory colony initiated with mosquitoes collected from the Archipelago of Fernando de Noronha, Brazil, less than a year earlier. Repellents were tested at 1% doses, and the protection data are expressed as mean ± SEM (n = 7, 5, 17, 21, 5, and 5, respectively). DEET-elicited protection against ZIKV-infected mosquitoes of the two groups was significantly lower than protection against noninfected mosquitoes (RecLab non-infected vs. ZIKV-infected, p = 0.0033; RecLab noninfected vs. FN ZIKV-infected, p = 0.0013, Mann-Whitney two-tailed test). Protection elicited by picaridin, albeit low, did not differ among the three groups (RecLab non-infected vs. ZIKV-infected, p = 0.8397; RecLab non-infected vs. FN ZIKV-infected, p = 0.3651, Mann-Whitney two-tailed test).

Next, we evaluated the sensitivity of the yellow fever mosquito to these two repellents by using a laboratory colony from Recife. Both DEET and picaridin had somewhat lower protection against 5- to 6-day-old, host-seeking *Ae. aegypti* females (Fig. 3) than observed for *Cx. quinquefasciatus* of similar age and using repellents at the same dose (Fig. 2). Despite its lower protection against the yellow fever mosquito than the southern house mosquito, DEET at 1% was a significantly better repellent (protection, 80.5 ± 4.3%) against *Ae. aegypti* than picaridin (protection, 51.8 ± 8.5%) was at the same dose (*n* = 4–6; *P* = 0.018, Mann-Whitney two-tailed test) (Fig. 3).

We then addressed the question of whether Zika virus infection would affect the response of the major Zika vector^3^, the yellow fever mosquito, to these two repellents. Mosquitoes infected with the Zika virus experienced a significant reduction in their response to DEET at 1% (*n* = 7 noninfected, *n* = 17 ZIKV-infected; *P* = 0.033, Mann-Whitney two-tailed test) (Fig. 3). The protection elicited by picaridin at 1%, albeit low, did not differ significantly between infected and noninfected mosquitoes (*n* = 4 noninfected, *n* = 21 ZIKV-infected; *P* = 0.84, Mann-Whitney two-tailed test). Likewise, yellow fever mosquitoes derived from a population from the island of Fernando de Noronha had a reduced response to picaridin (*n* = 5 ZIKV-infected; *P* = 0.365, Mann-Whitney two-tailed test) and significantly reduced response to DEET (*n* = 5 ZIKV-infected; *P* = 0.0013, Mann-Whitney two-tailed test) (Fig. 3). We surmised that the lower responses observed with ZIKV-infected mosquitoes could be caused by Zika infection itself and/or a normal aging process leading to a less-sensitive olfactory system.

**Figure 4:**
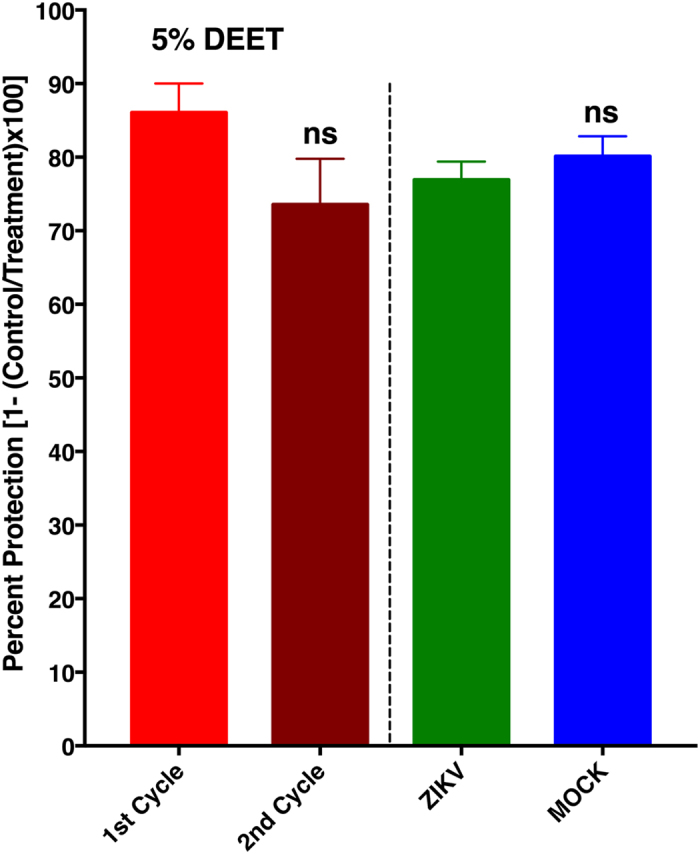
Comparative repellency activity elicited by DEET against blood-seeking mosquitoes in their first and the second gonotrophic cycles. Mosquitoes in the first cycle (red) were nulliparous. After the first behavioural measurements, mosquitoes were fed on defibrinated sheep blood and were tested again in their second gonotrophic cycle (brown). Two other groups of nulliparous mosquitoes were fed on blood either infected with the Zika virus (ZIKV) or with Vero cells (MOCK). Protection against older mosquitoes was somewhat lower, but not significantly different when tested at a 5% dose (p = 0.1159, 1st vs. 2nd cycle; two-tailed t-test). Likewise, protection against ZIKV-infected and mock-infected mosquitoes was not significantly different (p = 0.4083, two-tailed t-test). The data are expressed as mean ± SEM (n = 7–11).

Mosquitoes used for testing repellent efficacy in standard protocols must be nulliparous (never had a blood meal) and young, i.e., preferentially 5–7 days post-emergence^17^, or 5–10 days old^16^, according to WHO and EPA guidelines, respectively. We followed these guidelines in our tests, but mosquitoes naturally infected in field populations and capable of transmitting pathogens tend to be older^24^ than those used in efficacy tests following the WHO and EPA standard protocols. Although there is preliminary evidence suggesting that under laboratory conditions ZIKV can be transmitted from *Ae. aegypti* mothers to their offspring (vertical transmission)^25^, host-seeking female mosquitoes in the environment are more likely to transmit ZIKV after a second gonotrophic cycle. After an infective blood meal, the virus has to complete the extrinsic incubation period (EIP), i.e., the time required for the virus to pass through the midgut barrier into the mosquito’s hemocoel, invade the salivary glands, and replicate to a level that can be infective in the next blood meal. Meanwhile, mosquitoes complete the first gonotrophic cycle in a few days (e.g., 3–4 days for *Ae. aegypti* in Iquitos, Peru)^26^ and start host-seeking during the second gonotrophic cycle. Although EIP for ZIKV is not yet known, considering a lower estimate (10 days) for dengue virus (DENV)^24^, mosquitoes in the wild are unlikely to be ZIKV vectors before 12 days after emergence (given that they do not feed for the first time until they are about 2 days old). Therefore, the most dangerous mosquitoes are old individuals that already had at least one blood meal. Our initial tests and those for commercial repellents have been performed with mosquitoes too young (5–10 days old) for virus transmission. To test whether age and/or blood feeding affect mosquito responses to repellents, we measured repellency behaviour with the same group of mosquitoes during the first and second gonotrophic cycles. Responses of the southern house mosquito to 1% DEET in the first gonotrophic cycle (protection, 93.4 ± 4.1%) did not differ significantly from their response in the second gonotrophic cycle (protection, 95.6% ± 2.9%, *n* = 8, *P* = 0.92, Mann Whitney two-tailed test). At a lower dose (0.2%), there was a minor, albeit not statistically significant, reduction in the protection rate from the first to the second cycle (75.6 ± 5.4 vs. 73.4 ± 3.5%, *n* = 18, *P* = 0.46, Mann Whitney two-tailed test). Likewise, we observed a small reduction in protection against the yellow fever mosquito. Here, we tested a higher dose to enhance the protection rate. Protection in the first gonotrophic cycle increased from 80.5 ± 4.4% with 1% DEET (Fig. 3) to 86.1 ± 3.9% with 5% DEET (Fig. 4). Therefore, a higher dose of repellent is indeed desirable for protection against *Ae. aegypti*. Although a decrease occurred from the first to the second cycle, the reduction in protection was not significant (*n* = 7; *P* = 0.116, unpaired, two-tailed t-test) (Fig. 4). In short, older mosquitoes might have a reduced response to repellents, but this could be somewhat compensated by using higher doses.

Lastly, we compared the responses of ZIKV-infected and mock-infected mosquitoes to 5% DEET (Fig. 4). No significant differences were noted in the protection achieved with 5% DEET against mock-infected, ZIKV-infected, and noninfected mosquitoes of the same age group in the second gonotrophic cycle (Fig. 4). Similar experiments with 5% picaridin showed no significant difference (*P* = 0.98, Mann-Whitney two-tailed test) between mock-infected (protection, 61.4 ± 6.3% protection) and ZIKV-infected mosquitoes (protection, 59.3 ± 6.5%). Because no significant differences occurred in the responses to DEET or picaridin at 5% between mock-infected and ZIKV-infected mosquitoes, we analysed ZIKV-infected mosquitoes to determine whether they were indeed infected by the virus. Two pools of 10 engorged mosquitoes collected after the infection and two other pools of 10 mosquitoes each randomly collected 3 days post-infection (dpi) were analysed by quantitative reverse transcription polymerase chain reaction (RT-qPCR). Analysis of an aliquot of the ZIKV-tainted blood, which was supplied to mosquitoes for infection, confirmed that the virus titre was high (cycle threshold, Ct = 13) at the time of artificial feeding. The two pools of mosquitoes collected the day after infection generated Ct’s of 20 and 21, and both pools collected 3 dpi had a Ct of 19 thus confirming that mosquitoes were indeed infected. Lastly, ZIKV-infected mosquitoes used in the repellency assay comparing responses to repellents by ZIKV- vs. mock-infected mosquitoes were stored at −80 °C soon after the experiments, extracted in eight pools of up to 10 mosquitoes. Their Cts ranged from 15 to 17. We then concluded that ZIKV-infection per se does not significantly affect *Ae. aegypti* responses to the mosquito repellents DEET and picaridin. The acuity of the olfactory system of older mosquitoes may be somewhat reduced, but their lower sensitivity to repellents may be compensated by applying higher doses.

To compare the dose used in our experiment setup with normal applications of repellents, we quantified the amount of repellent applied to skin when using a commercial spray formulation. Spraying with Exposis Extreme^®^ (25% picaridin) 10 cm from the arm (n = 3) covered an area of approximately 42 cm^2^ with approximately 100.3 mg of the formulation, i.e., approximately 25 mg of picaridin. Thus, this spray covered the targeted area with 0.60 ± 0.04 mg/cm^2^, which corresponds to approximately a 6% dose in our laboratory assays.

The literature is dichotomous regarding the effect of virus infection on mosquito responses to repellents. Sindbis virus (SINV) infection has been shown to alter *Ae. aegypti* females’ responses to DEET^14^,^27^,^28^, but, on the other hand, no significant differences were observed in responses of DENV-infected mosquitoes^18^,^29^. There are significant differences in methodology between studies that prevent us from reconciling our data. First and foremost, the studies with DENV infection were conducted with nulliparous mosquitoes, which were intrathoracically inoculated with the virus. Thus, the DENV-infected mosquitoes were still in the first gonotrophic cycle, not to mention that there are concerns regarding the effect of thorax injections on the physiology of the animal. By contrast, the effect of SINV injection was studied using mosquitoes that received a blood meal and went through EIP. However, the age effect was not dissected by comparing mock-infected, SINV-infected, and untreated mosquitoes of the same age (and in the second gonotrophic cycle). They did compare virus-infected with mock-infected mosquitoes, but baseline data with uninfected mosquitoes were not reported. Except for only one report^18^, the effect of virus infection was examined in feeding behaviour and contact irritancy rather than examining DEET as a spatial repellent. Unfortunately, the study that examined responses of DENV-infected mosquitoes to DEET in a modified “arm-outside-cage” did not take into consideration the effects of age and a previous blood meal. The virus was inoculated by injection into the thorax, and only virus-infected and mock-injected mosquitoes were compared. Lastly, we cannot rule out the possibility that different populations (RecLab, Orlando, and Thailand) behave differently and, more importantly, that different viruses in our studies (SINV, DENV, and ZIKV) have different effects on the physiology of *Ae. aegypti* mosquitoes.

Our findings suggest DEET performs better as a spatial repellent than picaridin does, particularly against the yellow fever mosquito, when fresh samples of the two repellents were compared at the same dose. A full efficacy test for marketing purposes must include not only the initial efficacy, but also the complete protection time (CPT), i.e., the time between the application of a repellent and the first mosquito landing and/or probing^17^. From a pragmatic perspective, CPT does not matter if a repellent is not effective soon after its application. CPT determines for how long a repellent remains effective while it is being lost by abrasion, absorption, and evaporation. For high boiling point repellents like DEET and picaridin (b.p., 545°F = 285 °C and 296 °C at 760 mm Hg, respectively; PubChem) loss by evaporation is minimal. We observed, for example, that 4.62 ± 0.16% and 4.42 ± 0.45%, respectively, of samples of DEET and picaridin (n = 3 each) loaded on 10 ml beakers (height 3.4 cm; i.d. 2.2 cm) and left at 27 °C for 24 h were lost by evaporation. The major losses are by absorption through the skin^30^ and abrasion, which may lead to a significant reduction in the effective concentration of repellent remaining on the skin over time. Although higher doses of repellents lead to longer CPTs, it has been estimated that little gain in CPT is achieved by applying DEET at doses higher than 50%^31^. Based on peer-reviewed literature on efficacy tests of commercial formulations, DEET applications at a concentration of 20% or more had the best efficacy by providing up to 10 h of protection against *Aedes* bites^21^. By contrast, a recent evaluation by Consumer Reports placed a 20% picaridin-based and a 30% DEET-based product on the top of their list of recommended repellents with CPTs of 8 and 7.5 h, respectively, against *Aedes* mosquitoes. Albeit invaluable for consumers, this is not peer-reviewed evaluation, thus details, such as level of protection, are not known. When dealing with mosquitoes as a nuisance and at low population levels, a low level of protection may be acceptable, but in areas of ZIKV and other epidemics it is essential to prevent bites. It is conceivable that Consumer Reports considered in their overall assessment other properties (e.g., odour, ability to dissolve plastic) for which picaridin ranks better than DEET. Because of these properties and concern raised by patients, dermatologists recommend picaridin as a second-line agent, but concluded based on peer-reviewed literature that DEET demonstrated a strong and consistent ability to reduce mosquito biting relative to other repellents^32^.

Repellents have been widely tested and recommended for use based on their efficacy against young mosquitoes (5–10 days old) that never had a blood meal, but the most dangerous *Ae. aegypti* mosquitoes in the environments are older mosquitoes that have lived long enough after a blood meal for the virus to complete its extrinsic incubation period. Here, we show that *Ae. aegypti* at the age they can be infective seem to be less sensitive to repellents. Fortunately, applying higher doses of DEET compensated for the age-dependent reduced olfactory acuity. Currently, there are approximately 120 repellent formulations registered with the EPA for direct application to human skin and that contain 4–99% DEET^33^. Our findings suggest that wearing lower doses of repellents (e.g., 7% in commercial products), albeit useful against young, non-infective, nuisance mosquitoes, it might not be an effective measure for preventing virus transmission by older mosquitoes. On the basis of our findings, better protection may be achieved with formulations containing at least 30% DEET, which is nearly equivalent to 5% in our tests (Fig. 3). We are also cognizant of the suggestion that little gain in CPT may be achieved with doses higher than 50%^31^.

In conclusion, it seems that two-decade-old advice on using repellents remains mostly valid today with a caveat: “For a couple of hours in the backyard, a product containing 10 to 30% DEET should provide adequate protection. For a camping trip or a long hike, a 40 to 50% DEET preparation may be necessary”^34^. In light of our findings, it seems more appropriate to revise this advice and limit the lower doses to 30% DEET for those living or travelling to areas with ZIKV outbreak.

## Materials and Methods

### Mosquitoes

Two laboratory colonies of *Cx. quinquefasciatus* were used for repellency assays. The Davis laboratory colony originated from mosquitoes collected in Merced, California in the 1950s. The original “Merced colony” has been maintained in the Kearney Agricultural Center (KAC), University of California by Dr. Anthon Cornel. The laboratory colony used in this study was derived from the original colony kept at KAC and has been maintained at Davis for approximately six years under a photoperiod of 12:12 h (L:D), 27 ± 1 °C, and 75% relative humidity. We refer to it as the “Davis colony” to reflect the fact that it is a branch of the “Merced colony” kept at UC Davis. The Recife laboratory colony of *Cx. quinquefasciatus* (CqSLab) originated from eggs collected in Peixinhos, a neighbourhood of Recife, Brazil in 2009. Two colonies of *Ae. aegypti* mosquitoes were used. A laboratory colony, referred to as RecLab, was initiated from mosquitoes collected in Graças, a neighbourhood of Recife, Brazil in 1996. The FN colony originated from mosquitoes collected in the Archipelago of Fernando de Noronha, a district of Pernambuco state in 2015^35^. RecLab and FN colonies have been kept under a photoperiod of 12:12 h (L:D), 26 ± 2 °C, and 65–85% relative humidity in the insectary of the Department of Entomology, FIOCRUZ-PE.

### Chemicals

DEET (PESTANAL^®^ analytical standard grade, 99.5%) was acquired from Sigma-Aldrich (catalogue number, 36452–250 MG). Picaridin was a gift from Dr. Kamal Chauhan (USDA, ARS, Beltsville). Purity was estimated to be approximately 99% by gas chromatography/mass spectrometry GC-MS (5973 Networ, Agilent) and nuclear magnetic resonance, NMR (VNMRS600, Varian, Department of Chemistry). ^1^H NMR (600 MHz, CDCl3): δ 4.90 (m, 1H), 4.59 (br, 1H), 4.14 (br, 1H), 3.85 (br, 1H), 3.73–3.49 (m, 2H), 2.84 (dt, J = 13.24, 2.64 Hz, 1H), 2.08–1.54 (m, 10H), 1.35 (dd, J = 12.93, 6.25 Hz, 3H), 1.04 (dt, J = 10.37, 7.44 Hz, 3H). Our synthetic sample was compared by using GC-MS to a sample extracted from a commercial repellent, Exposis Extreme^®^ (25% picaridin), which was acquired from a supermarket in Brazil. The GC compartment was equipped with an HP-5MS capillary column (30 m × 0.25 mm; 0.25 μm), and the oven was operated at 70 °C for 1 min then the temperature was raised to 270 °C at a rate of 10 °C/min, and held at the final temperature for 10 min. Picaridin eluted at 13.64 min and gave the following spectrum: 128.1 (base peak), 41.1 (17), 57.2 (22), 84.1 (41), 156.1 (6), 184.2 (28), 229.2 (1%). Stock solutions (10%_m/v_) and their dilutions (5, 1, and 0.2%) were prepared in HPLC grade hexane (Fisher Scientific) and kept at −20 °C until use. Aliquots of 200 μl were applied to the edge of filter paper rings (treatment), and control rings were loaded with 200 μl of hexane. To avoid confusion, control and treatment filter paper rings were marked with two and three staples, respectively.

### Behaviour measurement

The surface landing and feeding behavioural assay has been described in detail elsewhere^20^. In short, a two-choice arena was constructed using an aluminium collapsible field cage (30.5 × 30.5 × 30.5 cm) with green polyester cover (Bioquip) to house at least 50 host-seeking female mosquitoes for each repellency test. The mosquito cage was attached to a frame that supported a wood board (30 × 30 × 2.5 cm) and held two Dudley bubbling tubes (painted internally with black glass ink) and two syringe needles separated from each other by 17 cm (centre to centre). The Dudley tubes were placed on a transverse plane at the middle line of the wooden board. One side of the mosquito cage was prepared with a red cardstock having openings to allow the Dudley tubes and syringe needles to protrude inside of the mosquito cage by 5.5 and 4 cm, respectively. The distance between each Dudley tube and syringe (8 mm) was just enough to hold dental cotton rolls. Insect pins placed 1.8 cm above the syringe needles held filter paper rings (width 4 cm; 25 cm; overlapped 1 cm for stapling), which served as a spatial repellent source (and control). Once defibrinated sheep blood (100 μl) was loaded on dental cotton rolls, carbon dioxide started to flow at 50 ml/min from each needle, and water at 38 °C circulated inside the Dudley tubes. The two choices (Fig. 1) differed only by a curtain of repellent at the side with the treatment filter paper ring. A zipper was sewn to the top surface of the mosquito cage to allow access to load cotton rolls, place rings, and aspirate mosquitoes, if needed. The surface opposed to the wooden board had an opening for a camcorder to document the experiments. Repellent assays with *Cx. quinquefasciatus* were run during the scotophase and using infrared light for recording. Repellent tests with *Ae. aegypti* were performed during the photophase and thus recorded with room light. To avoid interference, the experimenters left the room soon after each trial started and measured the responses by a combination of inspection at the end of the experiments and analysing the recordings. Repellence tests with *Ae. aegypti* were also monitored in real time with Cube+ cameras (Polaroid) inside of the room and iPhones (Apple) outside of the room. Behavioural data were generated from three batches of ZIKV-infected mosquitoes in concurrent observations (n = 5–21 replicates) for each experiment.

### Virus strain and artificial feeding

The ZIKV used for mosquito infection was a strain derived from a patient with maculopapular rash in Pernambuco, Brazil, during the outbreak of 2015. This strain, named ZIKV/*H. sapiens*/Brazil/PE243/2015, was sequenced in full-length and deposited in GenBank under the KX197192 accession number^36^. After isolation and passage of the virus on *Ae. albopictus* C6/36 cells, viral stocks were produced in Vero cells and stored at −80 °C until use. Three hundred thousand cells per ml were seeded in culture flasks (bottom area, 75 cm^2^) 24 h prior to ZIKV viral stock inoculation. After 24 h, an aliquot of ZIKV (500 μl) was used to infect Vero cells. Cells were incubated at 37 °C in a CO_2_ incubator for one hour to allow virus adsorption, and after that Minimum Essential Media (MEM) supplemented with 2% foetal bovine serum and 1% penicillin/streptomycin was added. Flasks containing infected and noninfected cells were then incubated at 37 °C and monitored daily until the appearance of cytopathic effects. Four to five days after ZIKV inoculation in Vero cell, flasks containing infected and noninfected (negative control) cells were subsequently frozen at −80 °C and thawed to 37 °C twice, and then mixed with defibrinated rabbit blood in a 1:1 proportion. Flasks containing noninfected Vero cells were used as negative controls (mock-infection). We generated three batches of ZIKV-infected *Ae. aegypti* mosquitoes for this study. Seven to ten-day-old female mosquitoes, kept on 10% sugar, were starved for 18 h prior to artificial feeding. Mosquitoes were allowed to feed on ZIKV-infected blood for 90 min, as detailed elsewhere^37^. Engorged mosquitoes were transferred to another cage and kept in a containment insectary at the Department of Entomology, FIOCRUZ-PE under a photoperiod of 12:12 h (L:D), 26 ± 2 °C, and 65–85% relative humidity.

### RNA extraction and virus detection

Prior to and after behavioural assays, mosquitoes were tested to verify whether ZIKV infection was successfully achieved. Briefly, mosquitoes were pooled in a maximum of ten individuals per 1.5 ml microtube and ground in 250 μl TRIzol^®^ (Invitrogen). RNA extraction followed the manufacturer’s protocol. ZIKV detection was performed using the protocol established by the Centers for Disease Control and Prevention (CDC)^38^ and detailed elsewhere^37^.

### Repellent evaporation

An aliquot of 50–80 mg of a neat repellent (DEET or picaridin) was loaded on a tared 10-ml beaker to form a thin layer at the bottom of the beaker with a surface area of approximately 3.8 cm^2^. The amount of repellent was measured accurately with an analytical balance (Ohaus, GA110), which was then zeroed and the total weight recorded. The beaker was placed inside of a 500-ml larger jar prepared with a bed of desiccant (drierite anhydrous, WA Hammond Drierite Company) on the bottom, which was then placed inside an incubator for 24 h at 27 ± 1 °C. The percentage loss by evaporation was calculated by the loss of weight/amount of repellent x 100. Experiments were done in triplicate for each repellent.

### Estimation of repellent applied by spraying

A commercial repellent (Exposis Extreme^®^, manufacture date: 4/28/2015; lot number 15118 B5), acquired from a supermarket in Brazil, was used for this estimation. The composition, according to the label, was water, icaridin (25%), denatured alcohol, disodium ethylenediaminetetraacetic acid, antioxidants, thickener, and preservative. Repellent was sprayed in triplicate 10 cm away from a clean forearm, and the area covered was measured. Likewise, repellent was sprayed in triplicate into tared beakers, and the total weight measured immediately. The total amount of repellent delivered by spraying under the described conditions was 100.3 ± 5.9 mg (i.e., approximately 25.08 mg of picaridin) and covered an area of 42 ± 3 cm^2^ of the arm. To determine whether the label percentage was accurate, an aliquot of the repellent was sprayed into a tared beaker, the weight determined, and solutions of 1,000 and 100 ng/μl were prepared by dilution in hexane. The picaridin peaks obtained with this 100 ng/μl solution by analysis with both gas chromatography and GC-MS were compared with the peaks derived from a sample of the same concentration, which was prepared starting with the neat synthetic compound. This analysis confirmed that the nominal percentage on the label was accurate.

### Statistical analysis

Behavioural responses are expressed in terms of protection rate, following WHO and EPA recommendations. Thus, P % = [1 − (T/C)] × 100, where C and T were the number of mosquitoes responding to the control and treated (repellent) side of the arena. For *Cx. quinquefasciatus* the number of mosquitos remaining inside of each arena at the end of each trial was recorded as a positive response. For *Ae. aegypti,* responses were recorded as the largest number of mosquitoes inside each arena not necessarily at the end, but at any time during each 15-min trial. Percentage protections were calculated with Excel spread sheets for subsequent analyses with Prism7 (GraphPad, La Jolla, CA). Data that did not meet the assumption of normality (Shapiro-Wilk test) or could not be transformed to pass this test were analysed using the Mann-Whitney, two-tailed test.

## Additional Information

**How to cite this article:** Leal, W. S. *et al*. Does Zika virus infection affect mosquito response to repellents? *Sci. Rep.* **7**, 42826; doi: 10.1038/srep42826 (2017).

**Publisher's note:** Springer Nature remains neutral with regard to jurisdictional claims in published maps and institutional affiliations.
